# Biomechanics on Ultra‐Sensitivity of Venus Flytrap's Micronewton Trigger Hairs

**DOI:** 10.1002/advs.202405544

**Published:** 2024-09-11

**Authors:** Kejun Wang, Siyuan Chen, Guanyu Bao, Tao Sun, Junqiu Zhang, Daobing Chen, Lining Sun, Zhiwu Han, Chao Liu, Qian Wang

**Affiliations:** ^1^ Jiangsu Provincial Key Laboratory of Advanced Robotics Soochow University Suzhou 215123 P. R. China; ^2^ Key Laboratory of Bionic Engineering Ministry of Education Jilin University Changchun 130022 P. R. China; ^3^ The Institute of Technological Science Wuhan University Wuhan 430072 P. R. China

**Keywords:** bio‐inspired, mechano‐sensor, micro‐cantilever, trigger hairs, ultra‐sensitivity

## Abstract

Numerous plants evolve ingeniously microcantilever‐based hairs to ultra‐sensitively detect out‐of‐plane quasi‐static tactile loads, providing a natural blueprint for upgrading the industrial static mode microcantilever sensors, but how do the biological sensory hairs work mechanically? Here, the action potential‐producing trigger hairs of carnivorous Venus flytraps (*Dionaea muscipula*) are investigated in detail from biomechanical perspective. Under tiny mechanical stimulation, the deformable trigger hair, composed of distal stiff lever and proximal flexible podium, will lead to rapid trap closure and prey capture. The multiple features determining the sensitivity such as conical morphology, multi‐scale functional structures, kidney‐shaped sensory cells, and combined deformation under tiny mechanical stimulation are comprehensively researched. Based on materials mechanics, finite element simulation, and bio‐inspired original artificial sensors, it is verified that the omnidirectional ultra‐sensitivity of trigger hair is attributed to the stiff‐flexible coupling of material, the double stress concentration, the circular distribution of sensory cells, and the positive local buckling. Also, the balance strategy of slender hair between sensitivity and structural stability (i.e., avoiding disastrous collapse) is detailed revealed. The unique basic biomechanical mechanism underlying trigger hairs is essential for significantly enhancing the performance of the traditional industrial static mode microcantilever sensors, and ensure the stability of arbitrary load perception.

## Graphical abstract

The Venus flytrap (Dionaea muscipula) possesses the ultra‐sensitivity to sense the external stimuli caused by prey for its conical morphology, multi‐scale functional structures, kidney‐shaped sensory cells, and combined deformation. The internal mechanical mechanism of the trigger hair is explored by biological research, finite element simulation, and original artificial hair.

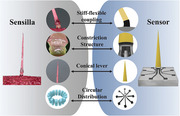

## 1 Introduction

Microcantilever‐based mechano‐sensors are widely used to precisely explore confined space under low visibility conditions,^[^ ^1^ ^]^ perform high‐quality measurement of free‐form surfaces,^[^ ^2^ ^]^ sense tiny changes in fluid disturbance, and detect biological and biochemical entities.^[^ ^3^, ^4^ ^]^ There are mainly two operating modes of microcantilever sensors: dynamic resonance mode and static bending mode.^[^ ^5^, ^6^, ^7^ ^]^ Dynamic resonance mode is mainly applicable to biochemical analyte detection through detecting frequency changes under surface biochemical binding.^[^ ^8^, ^9^ ^]^ Static bending mode, widely employed for physical and biochemical sensing, relies on the deformation and surface stress/strain under the mass or stiffness changes or the out‐of‐plane load caused by the touch or fluid disturbance.^[^ ^10^, ^11^, ^12^ ^]^ The traditional basic principle of the industrial static mode microcantilever sensor (ind‐SMMS) is based on the efficient transmission of the external stimuli from the lever to the bending root, where the signal transduction sensing‐unit distributes. Compared with the dynamic mode sensor needing externally added large excitation devices measuring the resonance frequency,^[^ ^13^, ^14^ ^]^ the ind‐SMMS does not depend on the excitation element. Concept of static bending mode has been a subject of extensive research efforts in recent decades due to its wider application. However, for the next generation intelligent equipment working in harsher environment, it is imminent to explore innovative ind‐SMMS for significant sensitivity enhancement, smaller size, and superior omnidirectional perception for the tiny mechanical signal.

Fortunately, the urgent functional enhancement principle of the innovative ind‐SMMS can be actually obtained from the plants such as Drosera, Arabidopsis, Utricularia and Venus flytraps (Figure S1, Supporting Information), which evolve microcantilever‐based ultra‐sensitive sensory hairs for satisfying prey capture or abiotic mechanical stimuli perception.^[^ ^15^, ^16^, ^17^, ^18^, ^19^, ^20^, ^21^ ^]^ Since the previous biological clearly indicates that, for detecting the out‐of‐plane mechanical signal, the synergistic relation of microcantilever and sensory cells makes the sensory hair as fascinating biological static mode microcantilever sensor (bio‐SMMS) maximizing the sensitivity and minimizing size.^[^ ^20^, ^22^ ^]^ As the most typical carnivorous plant, Venus flytraps, which live in nutrient‐poor habitats, can detect tiny tactile load caused by prey through touching mechanosensitive trigger hairs.^[^ ^18^, ^23^, ^24^ ^]^ In fact, the trigger hair is a typical multicellular hapto‐electric device that channels mechanical stress from the tip of the distal lever to the proximal podium where the sensory cells located in. The trigger hair can transmit tiny tactile stimuli caused by moving insect through the deformation of lever to the sensing cells that located at the root and can transduce the mechanical signal into action potential (AP) evoking the sudden trap closure.^[^ ^25^, ^26^, ^27^ ^]^ Then, the entrapped struggling insects would be further digested and absorbed by special hydrolases for meeting fundamental nutrient needs.^[^ ^28^ ^]^ Biological researches revealed that the micro‐scale ultra‐sensitive trigger hairs can elicit threshold APs for deflections of 2.9°, angular velocities of 3.4° s^−1^ and forces of 29 µN, efficiently detecting casually activity insects significantly smaller than ants.^[^ ^29^ ^]^ Definitely, the previous biological research clearly shows that the trigger hairs are fascinating bio‐SMMS with outstanding comprehensive performance. Absolutely, rather than superficial analogies, the design of the ultra‐sensitive ind‐SMMS inspired by the trigger hair is mainly based on the profound sensing biomechanical mechanism, which reflects the potential coupling between the ultra‐sensitivity and sensory‐related functional components such as multi‐scale structure, composite biomaterial and sensory cells.^[^ ^30^, ^31^, ^32^, ^33^ ^]^ However, while the extraordinary perception ability of the trigger hairs has been researched by biologists since the 19th century through behavior and physiological analysis, there is still an ultimate lack of understanding of the vital biomechanical mechanism.^[^ ^22^, ^34^ ^]^

In this paper, the sophisticated trigger hair of the Venus flytrap (*Dionaea Muscipula*) was researched.^[^ ^35^, ^36^ ^]^ Through a complementary combination of theoretical analysis, finite element modeling (FEM) simulation, and biomimetic fabrication, important biomechanical insights into the ultra‐sensitivity based on multiple strain concentration and positive local‐buckling strategies determined by unique structure and stiff‐flexible coupling materials were obtained. In addition, the vital trade‐off strategy between sensitivity and structural stability was revealed. The research presented in this paper provides innovative insights, demonstrating the trigger hair serves as an exemplary bio‐SMMS. It provides broad biologically inspired strategies not only for maximizing sensitivity and minimizing the size of ind‐SMMS by optimizing the distribution of the transduction sensing‐unit, but also for utilizing the micro‐structure and heterogeneous material.

## 2 Results and Discussion

### 2.1 The Distribution and Morphology of Trigger Hairs

**Figure 1:**
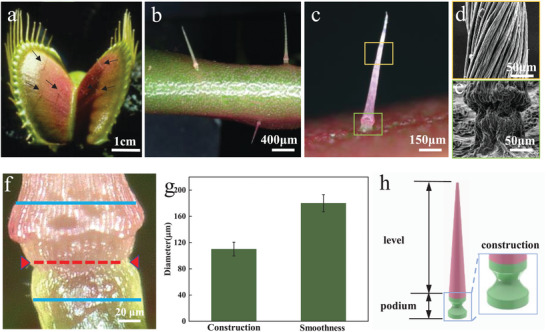
The morphology of the trigger hair. a) The overall morphology of Venus flytrap. b) Three trigger hairs on the upper epidermis of each of the flytrap's lobes. c) The morphology of the trigger hair: the long, pink distal lever, and the short, green basal podium. d) The morphology of the distal lever. e) The morphology of the proximal podium. f) The constriction structure at the proximal podium. g) The diameters of the constriction (red line in f) and smoothness (blue line in f) locations. h) The schematic diagram of the entire trigger hair.

The carnivorous Venus flytrap, which thrives in bright, humid, and nutrient‐deficient marshlands, has evolved specialized capture organs (i.e., snap traps) at the tip of each leaf for hunting animals to replenish their nutrition. The snap traps equipped with six touch‐sensitive trigger hairs, consist of two lobes (**Figure** **1**a). Each lobe features two long and one short trigger hairs, arranged in the shape of an isosceles triangle (Figure 1b).^[^ ^37^ ^]^ When wandering insects move on the angled open lobe, these insects will be detected by the accidentally touched trigger hairs that are deformed and then produce action potential.^[^ ^38^, ^39^ ^]^ Then, the flytrap would close rapidly and capture prey to respond the tiny mechanical stimuli. The statistical results reveal that the average total length of the long trigger hairs is 3115 ± 71.68 µm, while that of the short trigger hairs is 2800 ± 145.75 µm (Figure S2, Supporting Information). Detailed morphological research of trigger hairs further reveal that each multicellular trigger hair possesses two fundamental characteristics: a long, slender, pink distal lever with a straight stinger morphology and a short, light green proximal podium with a cylindrical feature (Figure 1c–e). Interestingly, Figure 1f illustrates that a slight constriction around the periphery of the trigger hair's podium. Here, the cell wall thickness is significantly reduced on the peripheral side. Consequently, as shown in Figure 1g, the diameter of the constriction (d≈115µ*m*, marked with a red dashed line) is smaller than the rest of the podium (marked with a blue solid line). Thus, the proximal podium can be more accurately described as a waist‐shaped rather than cylinder (Figure 1h).

**Figure 2:**
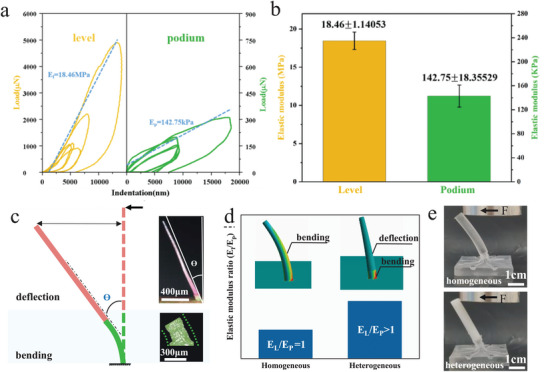
The deformation of the trigger hair. a) Two nanoindentation load‐displacement curves getting from podium and lever. b) Elastic modulus of Podium and lever, respectively c) The deflection of the distal lever and the bending of the proximal podium under lateral load. d) The ratios of elastic modulus between the lever (*E*_l_) and the podium (*E*_p_), along with the deformations of homogeneous and heterogeneous hairs during simulation, respectively. e) The deformations of homogeneous and heterogeneous artificial trigger hairs under lateral load.

As a typical mechano‐sensitive sensilla, the deformation of cantilever‐based trigger hair represents a critical issue that influences strain distribution and subsequently determines the sensitivity performance of these mechano‐sensilla. Perhaps, according to the classical theory of cantilever beams, the deformation behavior of cantilever beam is closely related to the elastic modulus of materials. Hence, we obtained the force‐distance curves in different regions through the nano‐indentation technique for investigating the elastic modulus of the distal lever and basal podium in fresh condition (**Figure** **2a**). Based on the Hertz model, the elastic modulus of the proximal podium, *E_p_*, and the distal lever, *E_l_*, are 142.75 ± 18.36 KPa and 18.46 ± 1.14 MPa, respectively (Figure 2b). To verify whether the difference in elastic modulus will bring beneficial combined deformation, here, we applied a tiny tactile load to simulate the stimuli from wandering animals for observing the deformation of trigger hair in a natural environment (Video S1, Supporting Information). The result clearly shows that, unlike the whole bending deformation observed in conventional cantilevers composed of homogeneous materials, the bending deformation in trigger hairs occurs only at the short basal podium. Meanwhile, while the distal long lever, devoid of strain energy undergoes significant deflection (Figure 2c). Consequently, the biological combined deformation strategy enhances the necessary strain, which evokes the action potential of sensory cells concentrated at the proximal podium. Meanwhile, the material configuration mechanism that determines the combined deformation of trigger hair was further investigated with an artificial cantilever beam. The elastic modulus of the flexible podium is 3Mpa and the elastic modulus of the stiff lever is 1Gpa, respectively (Figure S3, Supporting Information). Controlled experiments and finite element simulations reveal that biological‐related combined deformation, involving both bending and deflection, occurs only when the elastic modulus of podium is less than that of lever (Figure 2d,e). Therefore, the trigger hair consists of a stiff lever and a relatively flexible podium. And the impact of this unique combined deformation on the sensing performance of trigger hair will be further discussed in the subsequent research.

### 2.2 Multi‐Scale Structural Characteristic and Sensory Cells Distribution

**Figure 3:**
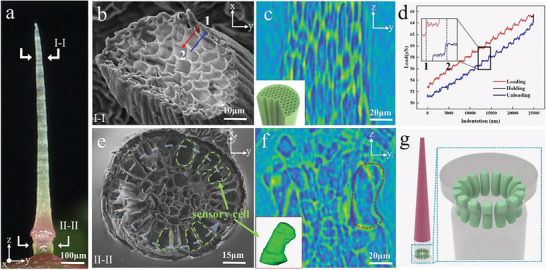
The structural characteristics and sensory cells distribution of the trigger hair. a) The external morphology of the whole trigger hairs. b) Cross‐sectional of the lever. c) Vertical‐section of the lever obtained by micro‐CT virtual slices. d) The load and displacement curves of the nanoindentation experiment. e) Cross‐sectional image of the podium. f) Vertical‐section of the podium obtained by micro‐CT scan and the kidney‐shaped sensory cells at the constriction structure. g) The schematic diagram of circularly arranged sensory cells at the constriction structure.

To further investigate the internal structure of the whole trigger hair (**Figure** **3**a), SEM and Micro CT were used to examine the characteristic of the distal lever and proximal podium. Figure 3b,c reveals that, unlike the commonly used totally hollow structure only achieving transport of water and nutrient in many functional hairs of carnivorous plants, the infrequent hexagonal honeycomb multi‐tubular structure was used by tactile‐sensitive trigger hairs (Figure S4, Supporting Information). The unique structural feature of the distal lever was also confirmed with bio‐nanoindentation experiments, which demonstrated that the applied load increases incrementally as the probe gradually pressed into the multi‐tubular structure of the trigger hair (Figure 3d). Each “platform” stage on the displacement‐load curve results from the movement of probe from one pierced wall surface to the next intact wall surface. Patently, the primary function of the tubular structure is to transport essential water and nutrition. However, more significantly, previous researches on elastic bucking have revealed that the honeycomb‐like cellular core also enhances the resistance to bucking caused by lateral and axial forces over that of a hollow cylinder of the same weight and morphology.

In examining the internal structure of the proximal podium, a ring of mechano‐sensory cells radially embedded around the constriction structure was found (Figure 3e,f; Figure S4, Supporting Information). Notably, all mechano‐sensory cells are distributed on the outer side away from the center axis (i.e, near the body surface).^[^ ^40^ ^]^ Previous biological research has demonstrated that mechano‐sensory cells, which generate critical action potentials (APs), are sensitive to tensile strain that takes on the responsibility of the opening of the mechano‐sensitive ion channels.^[^ ^22^ ^]^ When insects deflect the trigger hair, causing the podium to bend, the wall of one o strain‐sensitive sensory cell is stretched. Afterward, the tensile strain at the location of the sensory cell promotes the rise of cytoplasmic Ca2+ concentration. Once the Ca2+ concentration reaches a threshold value, an action potential (AP) is fired and then the flytrap is triggered to capture the prey.^[^ ^41^ ^]^ Utilizing 3D reconstruction software Mimics, we captured the 3D morphology of a single kidney‐shaped sensory cell, characterized by enlarged ends and a constricted middle (Figure 3f). Specifically, at the constriction structure, the sensory cells are arranged in a circular pattern around the hair's axis (Figure 3g). The concave side of the sensory cells faces outward, and their symmetric cross‐section aligns with the cross‐section of the constriction structure.

### 2.3 Ultra‐Sensitive Strategy of Micronewton Trigger Hairs

**Figure 4:**
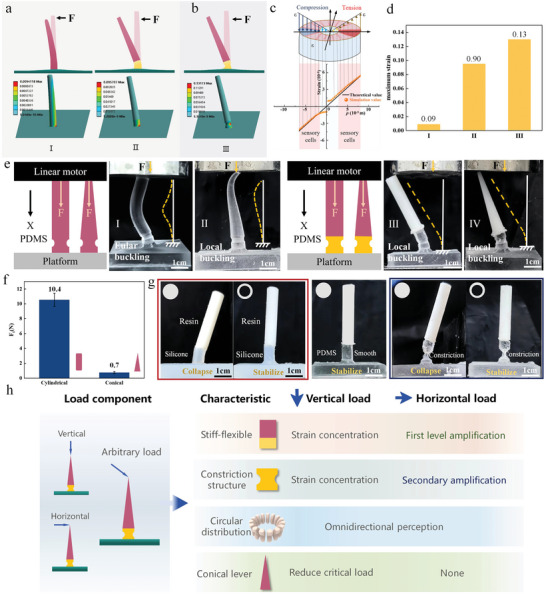
Biomechanical research of the artificial hair a,b) Deformation diagrams and finite element simulations of strain distributions of the artificial hair, (I) both the level and the podium were made of the stiff materials, (II) the level was made of the stiff material, and the podium was made of the soft material, (III) the level was made of the stiff material, and the podium was made of the soft material with a constriction structure. c) The distribution relationship between the strain and the distance from the axis at the constriction structure. d) The maximum strain of three different artificial hairs. e) Deformations of the trigger hairs with different materials and shapes under the same vertical load: (I) a homogeneous hair with cylindrical lever, (II) a heterogeneous hair with conical lever. (III) a heterogeneous hair with cylindrical lever, (IV) a heterogeneous hair with conical lever. f) The critical load of artificial trigger hair III and IV. g) (I) Compared with the trigger hair made of stiffer material (PDMS), the hair made of more flexible material (silicone gel) occurs “collapse”. Moreover, the hollow lever can avoid “collapse”. (II) Compared with the trigger hair with smooth structure, the hair with constriction structure occurs “collapse”. Moreover, the hollow lever (right) can avoid “collapse”. h) The influence of different characteristics on load components.

Why has the Venus flytrap evolved unusual tactile sensilla based on the coupling strategy of stiff‐flexible materials, which is totally contrary to the traditional engineered cantilever‐based mechano‐sensors composed of homogeneous materials? To address this question, we designed two cantilever beams with varying material compositions to simulate the impact of elastic modulus configurations on the sensitivity of trigger hair (**Figure** **4**a). Specifically, trigger hair I is composed of a homogeneous material, while the heterogeneous trigger hair II consists of a flexible podium and a stiff distal lever. Notably, for trigger hair II, the ratio of elastic moduli between the stiff lever and the flexible podium exceeds one order of magnitude. In addition, the elastic modulus of trigger hair I is equivalent to that of distal lever on trigger hair II. Figure 4c indicates the position of maximum tensile strain that is not determined by the composition strategy of the trigger hair, consistently aligns with the location of mechano‐sensory cells distributed at the periphery of the podium's cross‐section. According to the classical theory of the cantilever beam,^[^ ^42^ ^]^ the length of the distal lever, *l_l_*, is significantly greater than that of the podium's length, *l_p_*, (i.e., *l_l_* ≫ *l_p_*), consequently, the maximum strain, ε_ρ_, at any position on the podium of the trigger hair can be expressed by:

(1) $$\varepsilon_{\rho }\approx \frac{F_{h}\times L\times \rho }{E_{p}\times I_{c}}$$

Here, *F*_h_ is the horizontal load acting on the tip of distal lever, and *L* is the distance from the podium to the tip of the distal lever, which approximately equals the total length of the trigger hair. And *I*_c_ is the moment of inertia of the circular cross‐section at the constriction area. The ρ is the distance from the center of circle to target position. Equation 1 demonstrates a linear relationship between the ε_ρ_ and ρ. Furthermore, the maximum strain, denoted as ε_max_, of the entire trigger can be determined as follows: $\varepsilon_{\max}\approx \frac{F_{h}\times L\times R}{E_{p}\times I_{c}}$. Therefore, to ensure that the sensory cells always experience the maximum strain within the entire sensory structure, they are positioned at the podium's periphery. This arrangement effectively enhances sensitivity to weak signal (Figure 4c). Similarly, for a cantilever beam composed of homogeneous material with elastic modulus *E_l_*, the ε_max_ can be described as: $\varepsilon_{\max}=\frac{F_{h}\times L\times R}{E_{l}\times I_{c}}$. It is clear that the ε_max_ is consistently located at the position farthest from the tip, which is not determined by the composition strategy of cantilever. In addition, simulation results also indicates the edge at the section farthest from the tip provides the optimal location for sensory cells to consistently experience the maximum strain.

Moreover, Figure 3a further demonstrates that the maximum strain in trigger hair I, resulting solely from bending deformation, is significantly less than that in trigger hair II, which results from a combined deformation of bending and deflection. Obviously, compared to the stiff trigger hair I, the stiff‐flexible coupling strategy of trigger hair II significantly amplifies the maximum strain, consistently captured by one of the sensory cells (Figure 4d). This amplification occurs because the strain energy is efficiently concentrated on the flexible podium rather than being dispersed throughout the entire trigger hair. In fact, the maximum strain obtained by sensory cells can also be given by:

(2) $$\varepsilon_{\max}=\frac{F_{h}\times L}{E_{p}\times W}$$

Here, *W* is the section modulus of the cross‐section where the sensory cells are located, *E*_p_ is the elastic modulus of the podium. Equation 2 clearly demonstrates that the maximal tensile strain is solely determined by *E*_p_ and is unaffected by the elastic modulus *E*_l_ of the distal lever. For the cantilever composed of homogeneous material with specified elastic modulus *E*_l_, the $\varepsilon_{\max}=\frac{F\times L}{E_{l}\times W}$. Therefore, the ε_max_ obtained by sensory cell is significantly amplified due to the coupling strategy, this is because *E*_p_ is smaller than *E*_l_ (Figure S5, Supporting Information). In summary, the stiff‐flexible coupling strategy efficiently ensures that the Venus flytrap exhibits outstanding sensitivity, enabling it to detect tiny biological signals in harsh environments. We further designed trigger hair III, a stiff‐flexible coupling structure with a constriction at the position of the mechano‐sensory cells, to investigate the impact of this constriction structure on the sensitivity of the trigger hair (Figure 4b). Notably, the diameter of this constriction structure is smaller than those of other cross‐sections of the podium. Results clearly indicate that the maximum tensile strain in the trigger hair III is significant enhanced. Obviously, the constriction structure, exhibiting substantial stress concentration effects, leads to a secondary amplification of strain, thereby substantially increasing the sensitivity of trigger hair (Figure 4d). In fact, *W* in Equation 1 can be given by $W\;=\frac{\pi }{32}\;d^{3}$, further demonstrating that a moderate decrease in the diameter of the section across the sensory cells is another an effective strategy for enhancing the sensitivity by increasing ε_max_. Hence, regarding the horizontal load, *F_h_*, both the stiff‐flexible coupling design of the cantilever and the utilization of a constriction structure at the mechano‐sensory cells are crucial strategies for endowing the trigger hair with outstanding ultra‐sensitive performance. Additionally, the circular distribution of sensory cells further ensures that the trigger hair is sensitive to the tactile load from an arbitrary horizontal direction.

Inevitably, during the process of capturing prey, the tip of the trigger hair does not experience perfectly ideal horizontal tactile loads that would enable a single sensory cell to obtain the maximum tactile strain. Instead, it is subjected to arbitrary tactile loads that also include vertical component, *F_v_*. If the trigger hair was composed of homogeneous material, the vertical component would lead to undesirable Euler's buckling in the cylinder‐shaped hair and local buckling near the tip of the conical hair, making it difficult for sensory cells at the proximal podium to obtain the maximum tactile strain (Video S2, Supporting Information). This conclusion was confirmed by a compressive test on artificial hair made from homogeneous material (Figure 4e). Marvelously, even with the vertical component, the ingeniously coupling strategy comprising a long stiff distal lever, a short flexible proximal podium and a small constriction structure ensures the desirable local buckling occurs only at the position of sensory cells. This conclusion was further proved through a compressive test on bio‐inspired artificial trigger hair (Figure 4e). Notably, the distal lever is typically conical rather than cylindrical. Figure 4e indicates that artificial trigger hair with a conical lever also exhibits beneficial local buckling. Furthermore, for the conical and cylinder lever made of resin and possess the same elastic modulus. Comparative experiments clearly demonstrate that the vertical load threshold facilitating the local buckling of cylindrical lever (*F_t_* = 10.4 N) is significantly higher than that of a conical lever (*F_t_* = 0.7 N) (Figure 4f; Video S1, Supporting Information). Therefore, the unique conical characteristic of trigger hair is crucial for ensuring the sensitivity to vertical load (Figure S6, Supporting Information). Although the specific location of the maximum tactile strain under arbitrary vertical tactile component is not definite, the local buckling effectively ensures that maximum tactile strain is consistently localized to one of the mechano‐sensory cells distributed at the periphery of podium's cross‐section. Furthermore, considering the sensitivity to arbitrary horizontal component, it is crucial to recognize that the trigger hair possesses excellent ability of necessary omnidirectional perception for arbitrary tactile load with horizontal and vertical component.

### 2.4 The Trade‐Off Strategy Between Sensitivity and Structural Stability

Simply from the perspective of getting the optimal sensitivity, it is extremely beneficial for improving the sensitivity to decrease the value of podium's elastic modulus and the diameter of constriction structure as much as possible. However, from the aspect of pressure rod stability without an external load, excessively flexible podium and immoderately small diameter of constriction structure would lead a disastrous collapse of the trigger hair that caused by the Eular buckling of podium under axial load. And when there is not any external load applied on the hair, the gravity, *G_l_*, of distal lever is the axial load. According to the classical mechanics theory, the critical load, *F*_cr_, causing the buckling of podium can be given by: $F_{\mathrm{cr}}=\frac{\pi^{2}E_{p}I_{c}}{{\left(2l_{p}\right)}^{2}}$ that indicates that there is positive correlation between *F*_cr_ and *E*_p_. Obviously, the undue decrease of *E*_p_ (i.e, excessively flexible) would cause the disastrous collapse of hair due to *G_l_* > *F_cr_*. The above conclusion has been verified through the artificial trigger hair (Figure 4g). Specifically, the excessively flexible silicone with elastic modulus of 0.5 MPa and relatively stiff PDMS with elastic modulus of 3 MPa were used to design the proximal podium, respectively. The elastic modulus of distal lever is much higher than 3 MPa. The result shows that the podium composed of excessively flexible silicone presents unexpected buckling even without applying a load on the distal lever. For the biological trigger hair, the above severe buckling would cause the tactile strain at the sensory cell reaching the threshold to produce AP even without external tactile signal, which would further lead the catastrophic chaos of tactile perception. Similarly, because $I_{c}=\frac{\pi d^{4}}{64}$, the above equation further shows that the overly decrease of constriction structure's diameter would also cause the above unexpected disastrous collapse of hair due to *G_l_* > *F_cr_*. The above theoretical result has also been proved through the artificial hair (Figure 4g). In fact, it can be regarded that the buckling of the podium is caused by the over heavy distal lever. Therefore, to ensure the trigger hair contains more flexible podium and constriction structure with smaller diameter, the lightweight design (i.e., decreasing the *G_l_*) of distal lever is also vital to avoid the unexpected disastrous collapse. The comparative experiment clearly indicates the buckling of the flexible podium disappeared through using the lightweight distal lever with hollow tubular structure (Figure 4h). Notably, the lightweight design of distal lever without sacrificing necessary stiffness is vital for guaranteeing the sensitivity as well as avoiding the catastrophic chaos.^[^ ^43^, ^44^ ^]^ Fortunately, for reducing the mass of distal lever leading the pressure rod instability as well as maintaining high stiffness to the external stimuli, the trade‐off strategy between the weight and stiffness of distal lever has evolved through ingeniously utilizing the honeycomb hollow tubular structure that has also been widely employed by other animals for obtaining the high stiffness‐to‐weight ratio (Figure S7, Supporting Information).

### 2.5 Bio‐Inspired Design and Fabrication

**Figure 5:**
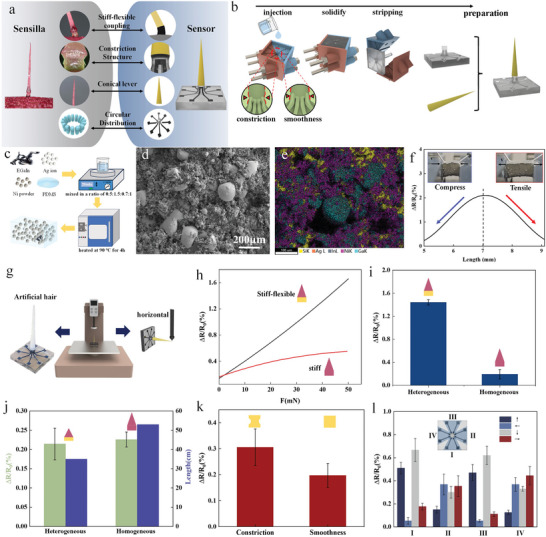
Biomimetic preparation and experimental verification. a) The mapping relationship between biological sensilla and artificial sensor. b) The preparations of artificial proximal podium and distal lever. c) The preparations of sensing units. d) The SEM image of the microstructure of sensing units. e) The EDS image of the element composition of sensing units. f) The variation curves of resistance change rate under tension and compression conditions. g) Construction of experimental platform. h) The fit curves between the resistance change rate and the load of the stiff‐flexible heterogeneous and the stiff homogeneous artificial hairs. i) The resistance change rate of homogeneous and heterogeneous artificial hairs under the same load (50µN). j) The resistance change rate of artificial hair with constriction structure and smoothness structure under the same load (5µN). k) The resistance change rate of a longer artificial hair with stiff podium and a shorter artificial hair with flexible podium. l) The resistance change rate of four sensing units under the same load in different directions.

In terms of bionic design, to further clarify the ingenious ultra‐sensitive mechanism of biological trigger hair, the diversified biological strategies for determining the sensitivity were simultaneously reflected in the artificial hair (**Figure** **5**a). Specifically, a stiff long distal lever and a flexible short proximal podium were designed to mimic the stiff‐flexible coupling strategy for achieving the strain amplification at the podium. The constriction structure was also designed on the podium of artificial hair for imitating the further (or secondary) strain amplification strategy based on the stress concentration. Meanwhile, the conical lever was designed to obtain a smaller critical vertical load. In our bio‐inspired samples, the composition of the essential flexible podium is the PDMS that cured on the 3D printed mold with precise multiple characteristics ensuring the sensitivity (Figure 5b; Figure S8, Supporting Information). In addition, the distal lever was directly prepared through resin material printing. And then, the podium is firmly bound with the 3D printed stiff lever through the ethyl α‐cyanoacrylate. Strain‐sensitive sensory cell, as the vital sensing element, achieves the transduction from mechanical signal to electrical signal. Similarly, the artificial sensing element based on the flexible piezoresistive material was designed to mimic the strain‐sensitive sensory cells. Here, both strain‐sensitivity and mechano‐electrical signal transduction are the essential functional similarity between the biological sensory cells and the piezoresistive material. Similar to the distribution of sensory cells, the multiple piezoresistive sensing elements are also circularly arranged at the outer side of the constriction structure for further demonstrating the omnidirectional‐sensitive strategy of trigger hair. The strain‐sensitive piezoresistive material is composed of PDMS, nickel powder, gain alloy, and nano silver conductive ink (Figure 5c). Figure 5d,e shows that the gain alloy is surrounded by nickel powder and presents a white spherical shape. The resistance change rate of piezoresistive material was analyzed by applying cyclic load through a displacement device. Figure 5f indicates that, under the tensile or compressive condition, the resistance of the piezoresistive material is much smaller than that in a relaxed state, which is caused by the decrease of distance between two conductive components in piezoresistive material (Figure S9; Video S4, Supporting Information).

From the aspect of sensitivity, a higher resistance change rate of the piezoresistive material represents a greater strain occur at the podium where the sensing elements distributed. To further verify the influence of diverse characteristics on the sensitivity of trigger hair, the corresponding horizontal load experiments were conducted on the bio‐inspired trigger hair, which involves all the characteristic of biological trigger hair (i.e., stiff‐flexible coupling, construction structure, conical lever and array distribution of sensory cells). And the response to external stimulation was characterized by resistance change rate (Δ*R*/*R*_0_) (Figure 5g). Differ from the all‐or nothing transients AP, although the resistance is continuous, simply from the aspect of sensitivity, a higher resistance variation of the piezoresistive material represents a greater strain occur at the podium where the sensing elements distributed, which is beneficial for signal perception. First, the Δ*R*/*R*_0_‐load curves clearly demonstrated that output of stiff‐flexible coupling trigger hair is significantly higher than that of the stiff artificial hair under the same mechanical stimulation (Figure 5h). For example, under the same horizontal load of 50 µN, the Δ*R*/*R*_0_ of stiff‐flexible coupling trigger hair is up to 1.44 ± 0.05, while the Δ*R*/*R*_0_ of stiff trigger hair is only ≈0.19 ± 0.08 (Figure 5i). The above results further indicate the necessity of stiff‐flexible coupling strategy for ensuring the sensitivity. Meanwhile, under the same load as well as output, the length (*l* ≈ 35 mm) of stiff‐flexible coupling trigger hair is shorter than that (*l* ≈ 53 mm) of stiff artificial hair (Figure 5j), indicating the stiff‐flexible coupling strategy can also efficiently achieve miniaturization without sacrificing sensitivity. Similarly, under the same horizontal load of 5 µ*N*, the Δ*R*/*R*_0_ (Δ*R*/*R*_0_ = 0.31 ± 0.07) of artificial hair with a constriction structure is higher than that (Δ*R*/*R*_0_ = 0.20 ± 0.05) of hair without such stress concentration structure (Figure 5k), further indicating the necessity of constriction structure for ensuring the sensitivity. Additionally, with the arbitrary loads applied on the tip of the artificial hair, there is always a sensing unit under maximum tensile strain obtaining the maximum rate of resistance change (Figure 5l). Hence, the above results further proved that the circular distribution strategy of sensing units effectively ensures the omnidirectional perception.

## 3 Conclusion

In summary, the biomechanical mechanism on the ultra‐sensitivity of Venus flytrap's trigger hair was revealed, which provides an excellent bio‐inspired strategy for enhancing the performance of ind‐SMMS. The results indicated that under the external load, the coupling design of the stiff lever and flexible podium realizes the first level amplification through efficiently concentrating strain energy caused by external stimulation on the podium where sensory cells are located. Then, the unique constriction structure at the sensory cells further amplifies the strain through stress concentration. Next, the conical feature of stiff lever significantly decreases the threshold of vertical load leading to the beneficial local buckling at the podium, which makes the trigger hair sensitive to the vertical component of arbitrary load. In addition, the tensile strain‐sensitive sensory cells were located at the outside of the podium and organized radially around the hair's axis, making one of the sensory cells always obtain the maximum tensile strain and guaranteeing the omnidirectional sensitivity of arbitrary load. Importantly, the hollow tubular structure maintaining a relatively high stiffness‐to‐weight ratio of distal lever effectively ensures the mechanical stability to avoid disastrous self‐buckling caused by lever's overweight. All above conclusions have been further verified through artificial bio‐inspired trigger hair. We also hold on that this work may provide essential biological reference value for researching the universal perception mechanism of sensory hairs.

## 4 Experimental Section

### Specimens

The B52 Venus flytraps were obtained from a reputable commercial supplier (https://chinese‐cp.jiyoujia.com). The Venus flytraps were kept in a 480 mL glassware with well‐drained peat moss to ensure the adequate nutrient. Meanwhile, the living condition of Venus flytraps was under an optimal room temperature of ≈22 °C.

### Characterizations

Scanning electron microscope (SEM) and Energy Dispersive Spectrum Analysis (EDS) studies were performed using a field emission SEM equipped with an EDS detector (SEM, EVO 18, ZEISS, Germany). In addition, the trigger hair dried by supercritical carbon dioxide (Drying equipment, NP‐5000‐300, CTECH Global Pte Ltd, America) was obtained by Micro‐CT (CT, Xradia 620 Versa, ZEISS, Germany). Then, the 3D structures were observed by the Dragonfly software (Object Research Systems (ORS) Inc, Canada) and the MIMICS software (Materialize Inc, Belgium).

### Nanoindentation Experiment

The Piuma Nanoindenter (Optics11, Netherlands) is mainly used for soft materials and biomaterials under the elastic modulus ranging from 5 Pa to 1 GPa. This nanoindenter uses a new type of fiber optic interferometric cantilevered spherical probe for indentation experiments, which is more suitable for measuring the soft material properties of Venus flytrap trigger hairs compared to AFM probes. The specific steps of the nanoindenter were as follows: the Venus flytrap trigger hair sample was placed on the microslide, the Poisson's ratio was set to 0.3. Under the Hertz model, the indentation depth was set to 5, 10, 20, 25 µm, respectively, the position of the tip of the trigger hair was adjust under the probe. Finally, the data viewer software was used to process the obtained data.

### Finite Element Simulation of the Sensory Hair

Two models based on the morphological characteristic of the Venus flytrap's trigger hair in SOLIDWORKS 2023 to simulate its response under out‐of‐plane tactile load were built. One of them had constriction structure and another didn't. The elastic modulus of the flexible podium is 100 MPa, Poisson's ratio is 0.3, and the elastic modulus of the stiff lever is 1 GPa, Poisson's ratio is 0.3. A lateral load of 500 µN was applied at the center of the circle on the upper surface of the lever. A horizontal component was applied on its upper surface and fixed its lower surface under different elastic moduli. All simulations were performed with geometric non‐linearity by Ansys Workbench 2023r1.

### Loading experiment

An artificial hair was fixed on the platform of tensile pressure testing machine (ZQ‐21A, ZHIQU, China) to measure the deformation of the trigger hair with diverse characteristic under horizontal and vertical components in the range of 2 and 50 N, respectively. The load was applied to the tip of the distal lever to obtain maximum strain in the podium. In addition, the buckling of artificial hair was also researched through applied vertical load.

### Biomimetic Preparation

Two molds were obtained by 3D printing (High temperature resistant resin, Wenext, China) and connected with four M5 bolts. A 5mm‐diameter circular channel was used for preparing the distal lever, and the protruding lines on the template surface were used to form grooves for filling the sensing material. Then, the configured Polydimethylsiloxane (PDMS) (Elastic modulus of 0.0004 to 0.0035 GPa) in a mass ratio of 10:1 (prepolymers: cross linker) was injected into the mold and heated at 90 °C for 4 h to solidify. Finally, connect the 3D printing lever with the demold podium to obtain the artificial trigger hair.

### Piezoresistive Material Preparation

The piezoresistive material was used as the signal transduction element. Initially, PDMS, nickel powder, gallium indium alloy, and nano‐silver conductive ink were weighed in a mass ratio of 1:1.5:0.5:0.7 and stirred at a speed of 1500 rpm for 10 min to ensure uniform mixing. Subsequently, the grooves in the podium were filled with piezoresistive material through an air pump. Finally, the artificial hair was place in a vacuum drying oven and heated at 90 °C for 4 h to obtain the solidified. The PDMS, nickel powder, gallium indium alloy, and nano‐silver conductive ink were purchased from Aladdin Chemical.

## Conflict of Interest

The authors declare no conflict of interest.

## Supporting information



Supporting Information

Supplemental Video 1

Supplemental Video 2

Supplemental Video 3

Supplemental Video 4

## Data Availability

The data that support the findings of this study are available in the supplementary material of this article.;
