# Supplementary material for: Biomechanics on Ultra‐Sensitivity of Venus Flytrap's Micronewton Trigger Hairs
Source: Adv Sci (Weinh). 2024 Sep 11;11(41):2405544. doi: 10.1002/advs.202405544 (PMC11538643; doi:10.1002/advs.202405544)
Supplement: Supplementary file 1 — Supporting Information [file ADVS-11-2405544-s002.docx]

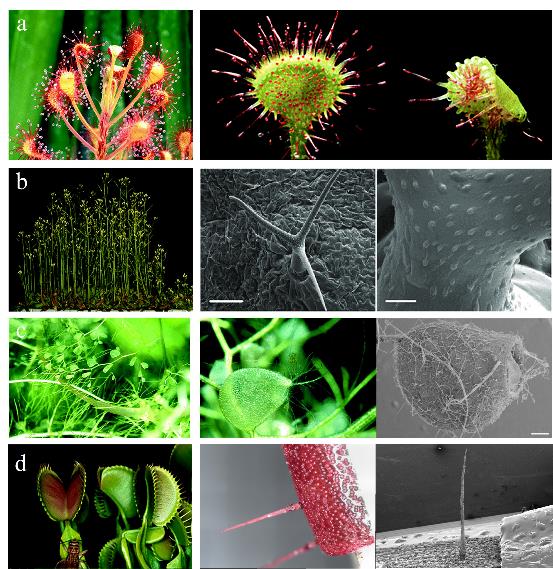


**Figure S1. Summary of plant hairs with sensory functions: a Drosera (Sundew), b Arabidopsis, c Utricularia (Bladderwort) d Venus flytraps.**

[1] J. Braam, *New Phytologist* **2004**, *165*, 373.

[2] H. Liu, L. H. Zhou, J. Jiao, S. Liu, Z. Zhang, T. J. Lu, F. Xu, *ACS Applied Materials & Interfaces* **2016**, *8*, 9755.

[3] K. Reifenrath, I. Theisen, J. Schnitzler, S. Porembski, W. Barthlott, *Flora - Morphology, Distribution, Functional Ecology of Plants* **2006**, *201*, 597.

[4] E. Saikia, N. F. Läubli, H. Vogler, M. Rüggeberg, H. J. Herrmann, I. Burgert, J. T. Burri, B. J. Nelson, U. Grossniklaus, F. K. Wittel, *Biomechanics and Modeling in Mechanobiology* **2021**, *20*, 2287.

**Figure S2. Statistical data on the length of the trigger hair’s distal lever (L_l_) and its proximal podium (L_p_).**

**Figure S3. The specific elastic modulus of the artificial trigger hair (the podium and lever is approximately equal to 3.0Mpa and 1.0Gpa, respectively).**

**Figure S4. The three-dimensional reconstruction diagram of circularly distributed sensory cells and hollow tubular structures.**

**Figure S5. The critical vertical load of the artificial trigger with different podium with different elastic moduli, the relationship between the magnitude of elastic modulus is: softer rubber＞PDMS＞silica gel.**

**Figure S6. The critical vertical load of artificial hair with different diameter on the tip (Ⅰ=2.2mm, Ⅱ=2.9mm, Ⅲ=3.6mm, Ⅳ=4.3mm, Ⅴ=5.0mm)**

**Figure S7. The reference of the hollow tubular structure to resist the impact load:**

[1] D. Hu, Y. Wang, B. Song, L. Dang, Z. Zhang, *Composites Part B: Engineering* **2019**, *162*, 21.

[2] Y. Li, B. Zhang, S. Niu, Z. Zhang, W. Song, Y. Wang, S. Zhang, B. Li, Z. Mu, Z. Han, L. Ren, *Acta Biomaterialia* **2022**, *147*, 91.

[3] J. Zhang, G. Tan, M. Zhang, D. Jiao, Y. Zhu, S. G. Wang, Z. Liu, D. Liu, Z. Zhang, *Journal of The Mechanical Behavior of Biomedical Materials* **2019**, *91*, 278.

[4] W. Yang, C. Chao, J. McKittrick, *Acta Biomaterialia* **2013**, *9*, 5297.

**Figure S8. Preparation process diagram of original artificial trigger hair.**

**
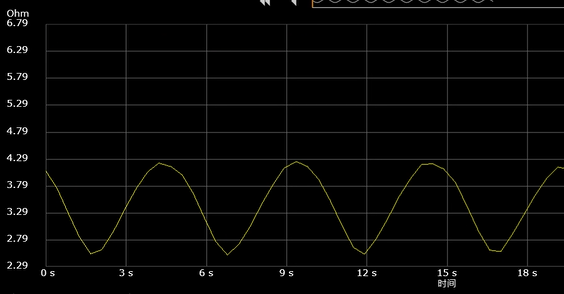
**

**Figure S9. The resistance change rate of the sensing material under tensile and compress condition.**
